# Altered proteome of a cystic fibrosis cell line model upon exposure to *Pseudomonas aeruginosa* biofilms

**DOI:** 10.1128/mra.01148-24

**Published:** 2025-10-20

**Authors:** Tia Rizakos, Jennifer Geddes-McAlister

**Affiliations:** 1 Molecular and Cellular Biology Department, University of Guelph 3653, Guelph, Ontario, Canada; Indiana University Bloomington, Bloomington, Indiana, USA

**Keywords:** biofilms, cystic fibrosis, *Pseudomonas*, proteomics

## Abstract

Cystic fibrosis is associated with disruption of a key ion channel responsible for maintaining lung health. Upon disruption, fluids build within the lungs, creating a favorable environment for bacterial invasion. In this study, we assessed altered proteome regulation of a cystic fibrosis cell line model in response to bacterial biofilms.

## ANNOUNCEMENT

Cystic fibrosis (CF) is an autosomal recessive disorder primarily caused by a mutation in the cystic fibrosis transmembrane conductance regulator (1). This mutation results in excessive water absorption, leading to impaired mucociliary clearance, activation and recruitment of immune cells to the lungs, and increased bacterial attachment to the epithelium causing inflammation and infection in the lungs (2–5). The Gram-negative bacterium, *Pseudomonas aeruginosa*, colonizes the lungs of people with CF (pwCF) as they age (6), occurring in approximately 85% of pwCF and being the predominant cause of morbidity and mortality (7, 8), resulting from bacterial adherence, biofilm formation, and resistance to innate defenses (9, 10).

In this study, we assessed the proteome response of neutrophils, a first line of defense against bacterial pathogens, toward *P. aeruginosa* biofilms within a CF cell line model (i.e., HL-60 cells with a genetic mutation in the F508 gene) (11). Our previous assessment of HL-60 cells exposed to *P. aeruginosa* biofilms provided a baseline for neutrophil remodeling upon biofilm exposure (12). For *P. aeruginosa*, the lab-associated (PA01; ATCC: BAA-47) strain was cultured from a single colony into Lysogeny broth (LB) (5 mL) at 37 °C with shaking (200 rpm) and normalized to an OD_600nm_ of 0.5. To induce biofilm formation, an aliquot (5 µL) of culture was grown statically in 200 µL of LB for 24 h at 37 °C; biofilm formation was confirmed via crystal violet staining (13). For neutrophils, a suspension cell culture model of HL-60 ∆F508 cells (gifted from Dr. Guoshun Wang) was maintained at 37 °C and 5% CO_2_ using Roswell Park Memorial Institute (RPMI) 1640 medium supplemented with 10% fetal bovine serum, 1% l-glutamine, and 1% antibiotic-antimycotic (Anti-Anti) for 5–15 passages (11). Cells were treated with fresh media containing 1.25% dimethyl sulfoxide every 2 days to promote neutrophil differentiation, collected by centrifugation (400 × *g* for 1 min), washed two times with phosphate-buffered saline, and resuspended in RPMI 1640 medium (without Anti-Anti) (11). To establish the co-culture model, ∆F508 neutrophils were incubated for 4 h at 37 °C and 5% CO_2_ with *P. aeruginosa* at a multiplicity of infection of 1:100 (neutrophils: PAO1 biofilms).

Samples were prepared in biological quadruplicate, and protein extraction was performed as we previously described (12, 14). Briefly, neutrophils from the co-culture were collected and pelleted at 400 × *g* for 4 min followed by resuspension in 100 mM Tris-HCl (pH 8.5) with a proteinase inhibitor cocktail tablet, 2% sodium dodecyl sulfate, and cell lysis via probe sonication. Dithiothreitol (10 mM final concentration) and iodoacetamide (5.5 mM final concentration) were added, and proteins were precipitated (−20 °C overnight) followed by quantification by tryptophan release (15), trypsin/LysC digestion, and purification (16). Peptides were quantified at OD_205nm_, and 3 µg of peptides was loaded onto a 50 cm PepMap RSLC EASY-Spray column (75 µM inner diameter) filled with 2 µm C18 reverse-phase silica beads (Thermo Fisher Scientific) and electrosprayed with a linear gradient of 3–20% Buffer B over 2 h using an Easy-nLC 1200 liquid chromatography system (Thermo Fisher Scientific) coupled to an Orbitrap Exploris 240 hybrid quadrupole-orbitrap mass spectrometer (Thermo Fisher Scientific). The mass spectrometer was operated in data-dependent acquisition mode. Raw data files were processed with MaxQuant (version 1.6.17) (17, 18) against the reference *Homo sapiens* proteome (82,492 sequences; December 2022), and *P. aeruginosa* (PAO1) proteome (5,564 sequences; December 2022). Parameters were set to default expect, enabling label-free quantification, match between runs, and minimum of two peptides for protein identification. Data were analyzed and visualized using Perseus (version 1.6.2.2) (19) with valid values (three out of four replicates, in at least one group), imputation (normal distribution, width of 0.3 and a downshift of 1.8), and normalization (subtract median from column). Protein abundance significant differences were determined by Student’s *t*-test (*P* value ≤ 0.05) corrected with the Benjamini-Hochberg false discovery rate (FDR) (FDR = 0.01) and *S*_0_ = 1 (20).

**Fig 1:**
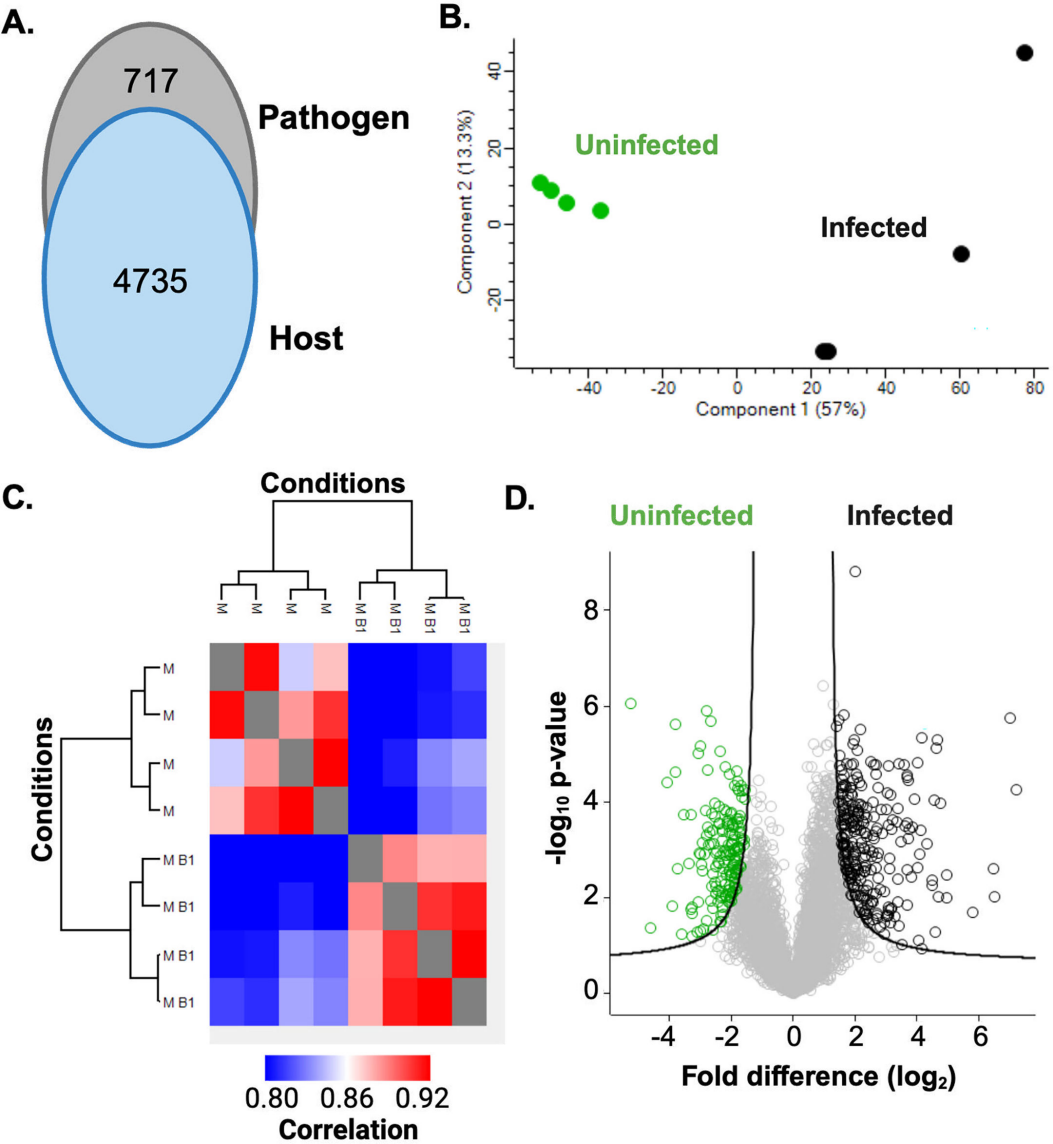
Proteome profiling of ∆F508 neutrophils co-cultured with *P. aeruginosa* biofilms. (**A**) Number of proteins identified from each biological system. (**B**) Principal component analysis. (**C**) Heat map of hierarchical clustering by Euclidean distance. M = ∆F508 uninfected; M B = ∆F508 infected. (**D**) Volcano plot. Student’s *t*-test *P* value < 0.05, FDR = 0.01, *S*_0_ = 1. Performed in biological quadruplicate.

We identified 4,735 host proteins and 717 bacterial proteins (Fig. 1A). A principal component analysis showed distinct clustering between infected and uninfected ∆F508 neutrophils (component 1, 57%) and we predict clustering of biological replicates based on similarities among uninfected samples and separation upon infection, indicating possible proteome remodeling during exposure to bacterial biofilms (component 2, 13.3%) (Fig. 1B). A heat map for hierarchical clustering by Euclidean distance showed replicate reproducibility at 89.3% (∆F508 neutrophils-only) and 90.0% (∆F508 neutrophils co-cultured with *P. aeruginosa*) (Fig. 1C). A volcano plot defined 175 proteins with a significant increase in abundance for uninfected ∆F508 neutrophils compared to 271 proteins with a significant increase in abundance during infection, including proteins critical for translation and host defenses (21) (Fig. 1D). Overall, this study provides new insights into CF-associated alterations in cell function that influence the interaction with bacterial pathogens during infection.

## Data Availability

The mass spectrometry-based proteomics data are available through PRIDE Proteome Exchange with accession number: PXD056790.
